# Influence of Sterilized Human Fecal Extract on the Sensitivity of *Salmonella enterica* ATCC 13076 and *Listeria monocytogenes* ATCC 15313 to Enrofloxacin 

**DOI:** 10.3390/antibiotics2040485

**Published:** 2013-12-02

**Authors:** Youngbeom Ahn, Ryan Stuckey, Kidon Sung, Fatemeh Rafii, Carl E. Cerniglia

**Affiliations:** Division of Microbiology, National Center for Toxicological Research, U.S. Food and Drug Administration, Jefferson, AR 72079, USA; E-Mails: rstuckey@uams.edu (R.S.); kidon.sung@fda.hhs.gov (K.S.); fatemeh.rafii@fda.hhs.gov (F.R.); carl.cerniglia@fda.hhs.gov (C.E.C.)

**Keywords:** fluoroquinolone, enrofloxacin, sterilized human fecal extract, *Salmonella*, *Listeria*

## Abstract

There is much debate on whether continuous exposure of commensal bacteria and potential pathogens residing in the human intestinal tract to low levels of antimicrobial agents from treated food animals pose a public health concern. To investigate antimicrobial effects on bacteria under colonic conditions, we studied resistance development in *Salmonella enterica* and *Listeria monocytogenes* exposed to enrofloxacin in the presence of fecal extract. The bacteria were incubated at 37 °C in Mueller-Hinton broth, with and without 0.01~0.5 μg/mL enrofloxacin, in the presence and absence of sucrose, and with 1% or 2.5% filter-sterilized fecal extract, for three passages. In the second and third passages, only the bacteria incubated in the media containing sterilized fecal extract grew in 0.5 μg/mL of enrofloxacin. Fecal extract (1% and 2.5%) decreased the sensitivity of *S. enterica* to enrofloxacin in the medium containing the efflux pump inhibitors reserpine and carbonyl cyanide-*m*-chlorophenylhydrazone (CCCP) and affected the accumulation of ethidium bromide (EtBr) in this bacterium*.* Enrofloxacin (0.06 µg/mL) and fecal extract altered the composition of fatty acids in *S. enterica* and *L. monocytogenes.* We conclude that fecal extract decreased the susceptibilities of *S. enterica* and *L. monocytogenes* to concentrations of enrofloxacin higher than the MIC and resulted in rapid resistance selection.

## 1. Introduction

The human intestinal tract is colonized by more than a thousand species of bacteria [1]. These bacterial species comprise what is known as a microbiota within the intestine and contribute to the normal functioning and overall health of the host [2]. The microbiota aids in digestion of food and metabolism of drugs and nutritional supplements, and contributes to various other functions, including metabolism, angiogenesis, enteric nerve function, and immunomodulation [3]. It also prevents colonization of the intestinal tract by foreign pathogenic bacteria and proliferation of pathogenic commensals through competition for space and nutrients [2]. Colonic microbiota may come in contact with antibiotics used for treatment and prophylaxis of infection and also with residual amounts of antibiotics in the products from food animals treated with antibiotics [4]. The U.S. Food and Drug Administration (FDA) evaluates antibiotic residue levels in food products for toxicological effects as well as how they impact the human intestinal microbiota [5,6].

Fluoroquinolones (enrofloxacin, norfloxacin, and ofloxacin) have been detected in tissues of chicken muscle and feather meal [7,8]. Enrofloxacin has been evaluated by the Joint Expert Committee on Food Additives (JECFA) and the European Medical Agency (EMA) has set the acceptable daily intake (ADI) values for enrofloxacin and ciprofloxacin at 2.3 μg/kg and 6.2 μg/kg of body weight, respectively [9,10]. It is not known if the ingestion of residues of antimicrobial agents in foods from treated animals is a public health risk and has the potential to influence changes in the antimicrobial sensitivity of the members of the colonic microbial community in the gastrointestinal environment. The colonic microbial community is exposed to a complex mixture of various substances resulting from digested and undigested food, ingested drugs or chemicals and their metabolites, microbial by-products, and various liver and intestinal secretions, which collectively form the bulk of the fecal materials [11]. The influence of this complex mixture on the interaction of the bacteria with antimicrobial agents in the gastrointestinal environment is unknown. We have previously described the effect of human fecal extract on the sensitivity of *E. coli* to the veterinary antimicrobial enrofloxacin or on the ability of bacteria to develop resistance to this drug [12]. In order to ensure that this effect is not restricted to one bacterium, we have investigated the effect of fecal extract in altering sensitivity to enrofloxacin in gram-positive (*L. monocytogenes*) and gram-negative (*S. enterica*) colonic pathogenic bacteria [13,14] and confirmed that indeed the substances present in colonic conditions have an effect on resistance selection to antimicrobial agents.

In this study, we have compared the effect of a low concentration of enrofloxacin on the sensitivity of *S. enterica* and *L. monocytogenes* grown in media with different additives, including fecal extract. The MIC of enrofloxacin for *S. enterica* and *L. monocytogenes* grown in the presence and absence of sterilized fecal extract for three passages was measured and the effect of fecal extract on the survival and the kinetics of the growth of both species was evaluated. The effects of growth of strains with enrofloxacin and fecal extract on cell morphology, fatty acid composition and metabolic activities were evaluated. 

## 2. Results and Discussion

### 2.1. Growth Kinetics of *S. enterica* and *L. monocytogenes*

**Figure 1:**
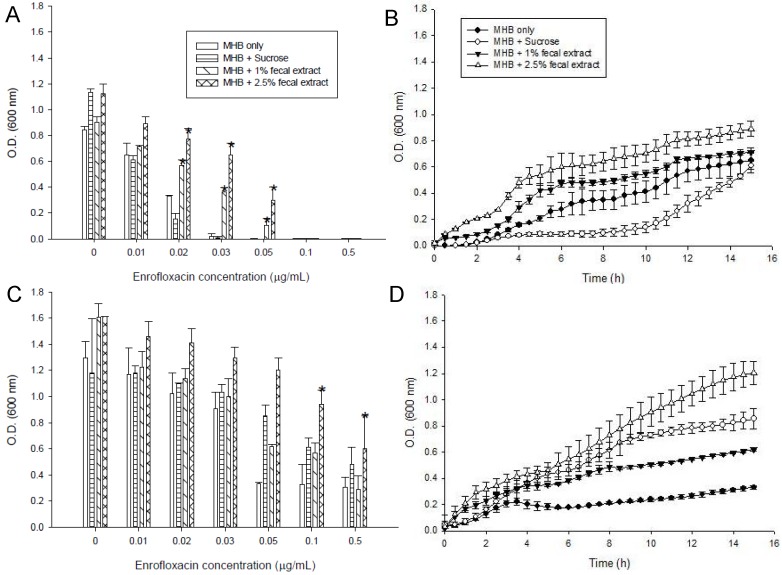
Effects of different concentrations of enrofloxacin on growth of *S. enterica* ATCC 13076 in media containing 5 mM sucrose, or 1 or 2.5% sterilized extract from a human fecal sample, (**A**) in the first passage; (**B**) Kinetics of survival with a sub-MIC concentration of enrofloxacin (0.01 μg/mL); (**C**) Maximum cell growth measured in the third passage in media with different supplements; (**D**) Kinetics of growth of *S. enterica* in 0.05 μg/mL of enrofloxacin measured in the third passage. Symbols represent averages of triplicates from three samples and error bars represent the standard deviations. * Indicates statistically significant differences from control (*p* < 0.05).

**Figure 2:**
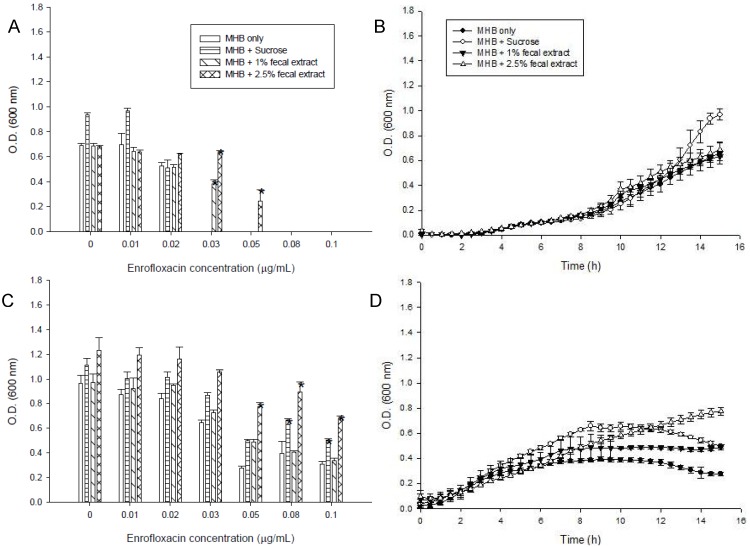
Effects of different concentrations of enrofloxacin on growth of *Listeria monocytogenes* ATCC 15313 in media containing 5 mM sucrose, or 1% or 2.5% sterilized extract from a human fecal sample, (**A**) in the first passage; (**B**) Kinetics of growth of *L. monocytogenes* in media with different supplements in 0.01 μg/mL enrofloxacin; (**C**) Maximum cell growth measured in the third passage in media with different supplements; (**D**) Kinetics of growth of *L. monocytogenes* in 0.05 μg/mL of enrofloxacin measured in the third passage. Symbols represent averages of triplicates from three samples and error bars represent the standard deviations. * Indicates statistically significant differences from control (*p* < 0.05).

The MIC of enrofloxacin for *S. enterica* (Figure 1A) and *L. monocytogenes* (Figure 2A) were 0.03 μg/mL in MHB media alone. Both 1% fecal and 2.5% fecal extract decrease the susceptibilities of the strains to enrofloxacin and *S*. *enterica* could grow with 0.05 μg/mL of enrofloxacin (Figure 1A). *S. enterica* growth rates in MHB supplemented with sucrose media varied in 12 h (Figure 1B). In the presence of sub-MIC (0.01 μg/mL) enrofloxacin, growth of *S. enterica* was higher in the medium supplemented with 1 or 2.5% sterilized fecal extract than in other media (Figure 1A,C). In the third passage, the bacteria that had survived in 0.01 μg/mL of enrofloxacin (sub-MIC) were used for inoculation. They grew well in all media containing up to the MIC (0.03 μg/mL) of enrofloxacin. Figure 1C compares the growth of *S. enterica* in different concentrations of enrofloxacin in MHB alone or MHB supplemented with sucrose and fecal extract in the first and third passages. Better growth was observed in MHB supplemented with 2.5% sterilized fecal extract (Figure 1D). 

Fecal extract also decrease the susceptibility of *L. monocytogenes* to the drug and this bacterium could grow with 0.05 μg/mL of enrofloacin (Figure 2A).The strains showed a slower rate of growth in the first 9 h of incubation in the media with 0.01 μg/mL enrofloxacin than in media without enrofloxacin (Figure 2B). *L. monocytogenes* grew well in all media containing up to the MIC (0.03 μg/mL) of enrofloxacin (Figure 2C,D). In the third passage, *S. enterica* and *L. monocytogenes* could grow in the higher concentration of enrofloxacin (0.5 and 0.1 μg/mL) in MHB with or without additives. Media supplemented with sterilized fecal extract and sugars also better supported the growth of *S. enterica* and *L. monocytogenes* in the third passage (Figure 1C and Figure 2C).

### 2.2. Comparison of the Sequences of the QRDR and PFGE

The QRDR primers were used to amplify 251 bp fragments from the cells grown in the wells containing MBH medium alone and those grown in the wells containing different concentration of enrofloxacin in the presence and absence of fecal extracts in the second and third passages. The 96 resulting PCR amplicons were sequenced and analyzed. The sequences of the QRDR from all of the *S. enterica* strains were identical, regardless of the level of sensitivity to enrofloxacin in the second and third passages (data not shown). 

To find out if the bacteria that survived in higher than the MIC concentrations of enrofloxacin had mutations in the genomic DNA, the PFGE profiles of 24 samples of each *S. enterica* and *L. monocytogenes* were compared for bacteria grown in MHB, and MHB supplemented with fecal extract, with and without 0.06 µg/mL of enrofloxacin. No differences were observed in the PFGE patterns of *S. enterica* and *L. monocytogenes* grown under different conditions (data not shown). 

### 2.3. Efflux Pump Inhibitors and Intracellular EtBr Accumulation in *S. enterica*

**Figure 3:**
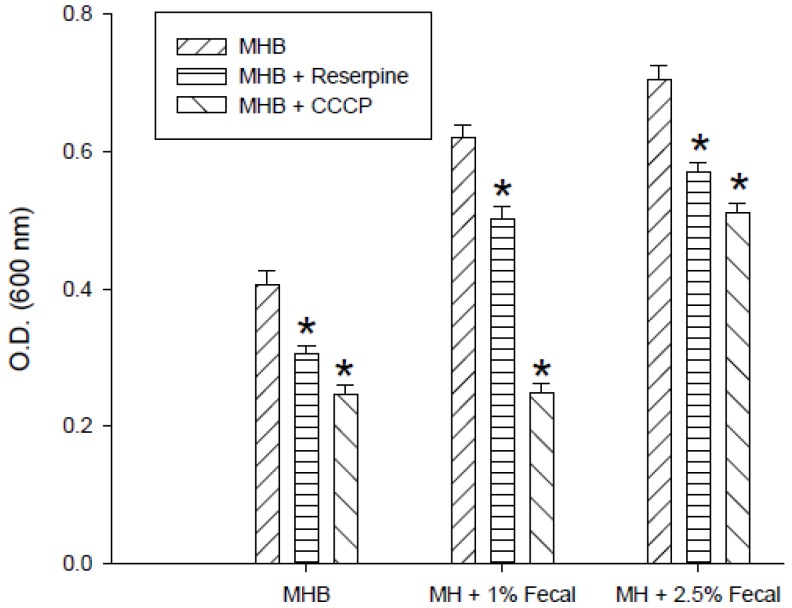
Effects of reserpine (33 µM) and carbonyl cyanide-*m*-chlorophenylhydrazone (CCCP) (10 µM) on the growth of *S. enterica* with 0.1 μg/mLenrofloxacin in Mueller-Hinton broth (MHB) containing 1% and 2.5% sterilized human fecal extract in the third passage. * Indicates statistically significant differences from control (*p* < 0.05).

The effect of fecal extract on decreasing the bacterial sensitivity to enrofloxacin by the efflux pump inhibitor CCCP (10 µM) or reserpine (33 µM) was examined in the medium containing 0.1 μg/mL enrofloxacin. The growth of *S. enterica* from the third passage in MHB with or without 1% and 2.5% fecal extract is shown in Figure 3. Reserpine and CCCP increased the sensitivity of bacteria to 0.1 μg/mL of enrofloxacin in MHB. Fecal extract (1% and 2.5%) decreased the sensitivity to enrofloxacin in the medium containing reserpine and CCCP. 

**Figure 4:**
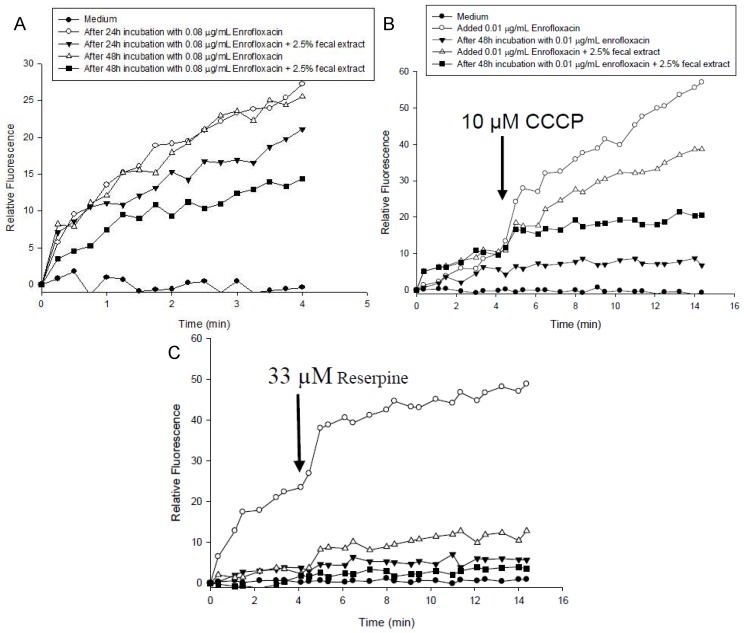
(**A**) Accumulation of ethidium bromide by *S. enterica* after 24 h and 48 h incubation with 0.08 μg/mL of enrofloxacin in MHB containing 2.5% sterilized human fecal extract; (**B**) CCCP (10 μM); and (**C**) reserpine (33 µM) were added at the time indicated by the arrow.

The kinetics of accumulation of EtBr in *S. enterica* grown with 0.08 μg/mL enrofloxacin in the medium with or without 2.5% fecal extract is shown in Figure 4A. EtBr accumulation was higher in *S. enterica* grown in the medium alone than with 2.5% fecal extract for 24 and 48 h (Figure 4B,C). Addition of CCCP (Figure 4B) and reserpine (Figure 4C) to *S. enterica* increased EtBr accumulation in the samples grown with medium only. 

### 2.4. Effect of Enrofloxacin and Fecal Extract on *S. enterica* and *L. monocytogenes* Morphology

**Figure 5:**
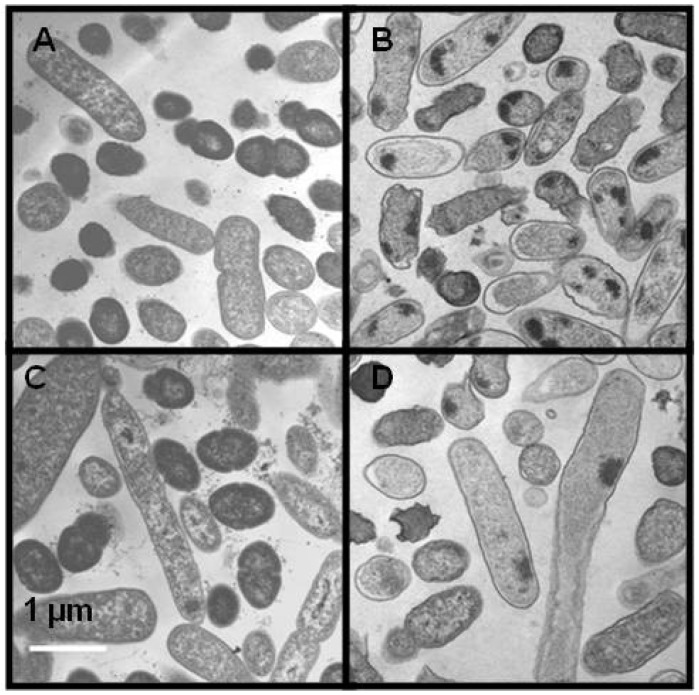
Transmission electron micrographs in the absence (**A**, **B**) or presence (**C**, **D**) of 0.05 μg/mL enrofloxacin for *S. enterica* (**A**, **C**) and *L. monocytogenes* (**B**, **D**). Scale bars indicate a length of 1 μm.

Enrofloxacin affected the morphology of the cells of both bacterial species. Some of the cells exposed to 0.05 μg/mL enrofloxacin were elongated in *S. enterica* (Figure 5C) and *L. monocytogenes* cells (Figure 5D) in comparison with the cells grown in the medium without enrofloxacin (Figure 5A,B). The increase in the number of elongated cells varied with the concentration of enrofloxacin. Presence of fecal extract in the media did not affect the morphology of either of bacterial species.

### 2.5. Effects of Sterilized Fecal Extract on Lipid Composition

**Figure 6:**
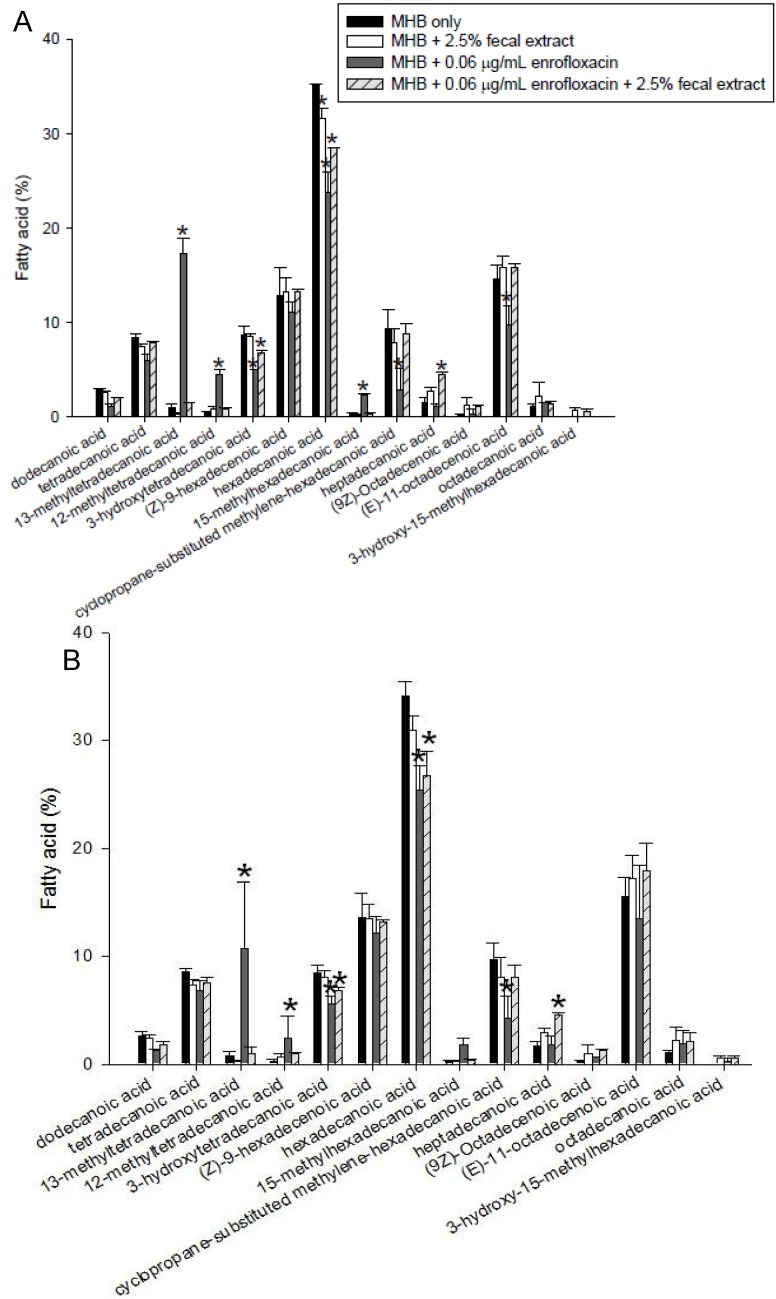
Fatty acid composition of lipids of *S. enterica* (**A**) and *L. monocytogenes* (**B**) grown with and without 0.06 μg/mL enrofloxacin in MHB with and without 2.5% fecal extract. The experiments were repeated three times using one human fecal sample and the figure shows the typical results. * Indicates statistically significant differences from control (*p* < 0.05).

The fatty acids of *S. enterica* and *L. monocytogenes* were analyzed in bacteria grown in MHB and MHB supplemented with fecal extracts, with and without 0.06 µg/mL of enrofloxacin (Figure 6). The major fatty acid in *S. enterica* and *L. monocytogenes* was hexadecanoic acid (C_16:0_), which constituted between 30%–40% of total fatty acids. In both species grown in media with 2.5% fecal extract, the percentage of saturated fatty acids decreased, while the unsaturated fatty acids increased in comparison with those grown in MHB alone. Also, in both bacteria grown with 2.5% sterilized fecal extract, the proportion of heptadecanoic acid (C_17:0_) increased, but that of hexadecanoic acid (C_16:0_) decreased. The presence of enrofloxacin also affected the fatty acid composition of bacteria grown in MHB alone. The percentage of 13-methyltetradecanoic acid (C_15:0__iso_) comprising <5% of the membrane increased in both strains grown with enrofloxacin. The percentage of 13-methyltetradecanoic acid in *S. enterica* grown in 0.06 μg/mL enrofloxacin increased to 15%–20% content. However, the presence of enrofloxacin did not have a substantial effect on the composition of fatty acids in the presence of fecal extract (Figure 6A,B).

### 2.6. Discussion

The human intestinal commensal and colonizing bacteria, some of which are potential pathogens, come into contact with antimicrobial agents in complex environments. In this study, we have investigated the potential of fecal extract in influencing enrofloxacin resistance development in two bacterial strains, *S. enterica* and *L. monocytogenes*. A low concentration of fecal extract enhanced the growth of *S. enterica* and *L. monocytogenes* in both MIC and sub-MIC concentrations of enrofloxacin in the first passage. Both bacteria allowed growth at 3–10 times the MIC of enrofloxacin in the second and third passages without inducing mutations in the QRDR region of *gyrA*. The fecal extract also decreased the effect of efflux pump inhibitors in enhancing the sensitivity of the cells to enrofloxacin and affected the composition and proportions of unsaturated and saturated fatty acids in the cells. Strains with reduced susceptibilities to enrofloxacin exhibited different metabolic patterns from wild types in both bacteria.

*S. enterica* and *L. monocytogenes* have been isolated from the fecal material of healthy individuals, and in the colon, along with other potential pathogenic commensally bacteria, come into contact with low to high concentrations of antimicrobial agents through ingestion of foods from treated animals and antimicrobials used for the treatment of infections [15]. Exposure to low levels of the drug in the colonic environment may potentiate the ability of strains to resist high concentrations of the drug.

Fecal extract has been shown to decrease antibiotic potency by various means, including decreasing interaction of the drug with the bacteria [12,16] and enzymatic degradation of the drug [17,18]. Neither *S. enterica* nor *L. monocytogenes* can degrade enrofloxacin, as evident by HPLC and LC/MS analyses of enrofloxicin before and after incubation with each bacteria with or without fecal extract (data not shown). To date, no specific gastrointestinal tract microbiota degrading fluoroquinolones has been identified [12,16]. The decrease in the susceptibility of the strains to the drug in the presence of fecal extract could be the result of modification of interaction of the cells with the drug by affecting the membrane or by binding to the drug.

Sub-MIC concentration of fluoroquionolone and other antimicrobial agents has shown to increase mutation rate. Golburg *et al*. [19] have shown that selection of resistant bacteria occurs at very low antibiotic concentrations (100 pg/mL of ciprofloxacin). The changes in the QRDR region of *gyrA* in many bacteria have been the reasons for fluoroquinolone resistance, which also has been associated with other changes in bacteria [20,21,22]. However, the QRDR region of *gyrA* of the resistant strains had the same sequence as that of the wild types. Other reasons for the bacterial resistance to fluoroquinolones have been decreases in permeability to the drug and enhanced activity of the efflux pump [14,23,24]. The observation that both CCCP and reserpine, which inhibit transport [15,25], affected EtBr accumulation in both resistant strains and affected bacterial growth with enrofloxacin indicates the involvement of the efflux pump in protecting both strains.

We have not investigated the presence of possible mutations outside of the QRDR region of *gyrA* or in other genes that are reported to contribute to fluoroquinolone resistance [20,21,22]. However, those mutations normally have been observed when bacteria have been exposed to high concentrations of fluoroquinolones, rather than the low concentrations used in our study. PFGE was performed to evaluate possible changes in the DNA pattern [26]. Although no differences in the PFGE patterns of resistant strains with their wild types grown with or without fecal extract were observed (data not shown), enrofloxacin affected the bacterial morphology, as evident from the negative staining and thin sectioned TEM of both *S. enterica* and *L. monocytogenes*. *S. ent/erica* exhibited an elongated shape. Previous studies have found that *E. coli* cells exposed to 0.01–0.1 µg/mL of enrofloxacin were elongated. The change in the structure of the cells was also reflected in the lipid composition of the membranes [12]. However, no difference in the morphology of bacteria was observed in bacteria grown with or without fecal extract.

When exposed to increasing concentrations of enrofloxacin, the plasma membranes of *S. enterica* and *L. monocytogenes*, grown in MHB alone, exhibited a lower percentage of saturated fatty acids and a higher percentage of unsaturated fatty acids. The difference observed in the fatty acid composition of isolates grown with or without fecal extract may relate to the differences in the fatty acid synthesis in strains. The fatty acid synthesis was not the subject of our study and need further investigation. Upon exposure to increasing concentrations of enrofloxacin of *S. enterica* and *L. monocytogenes* grown in 2.5% fecal extract, less significant changes in both saturated and branched fatty acids occurred. It is possible that the presence of the fecal extract inhibited the effect of the antibiotic on the plasma membrane due to the presence of currently unknown factors present in the extract and also acted as osmolytes. Similarly, the resistant strains could not grow well in GEN III MicroPlates (BIOLOG, Hayward, CA, USA) in the presence of some sugars and were inhibited in the well containing 8% NaCl, which may indicate less tolerance of resistant strains to osmotic pressure than the wild types. Interestingly, the fecal extract appeared to act as an osmoprotectant, since *L. monocytogenes* grown with fecal extract grew equally well in the presence and absence of high salt concentrations (data not shown).

In addition, *S. enterica* and *L. monocytogenes* grown in 2.5% fecal extract were capable of growing at higher concentrations of enrofloxacin than when grown in MHB alone. This could be due to the presence of numerous compounds that the bacteria used as nutrients, leading to increased growth of the bacteria [11]. Interestingly, both species grown with 5 mM sucrose were able to grow at higher concentrations of enrofloxacin than when grown with 1% fecal extract. Following exposure to low concentrations of enrofloxacin in MHB medium with or without supplements during the first 24 h, both *S. enterica* and *L. monocytogenes* could grew better in subsequent passages regardless of the treatment, indicating decreased susceptibility in a subpopulation of bacteria. 

## 3. Experimental

### 3.1. Bacterial Strain, Growth Media and Reagents

*S. enterica* ATCC 13076 and *L. monocytogenes* ATCC 15313 were grown on Trypticase Soy Agar with 5% Sheep Blood (BD, Franklin Lakes, NJ, USA) for 24 h and resuspended in sterile water to a concentration of 2 × 10^5^ colony forming units (CFU/mL) for inoculation. Sterile Mueller-Hinton broth (MHB) was prepared according to the manufacturer’s instructions. The fecal extracts were prepared according to the method described previously [12]. Briefly, the fecal suspensions were diluted with MHB to produce 10% and 25% (w/v) dilutions and sterilized by autoclaving at 121 °C for 30 min. The sterile fecal suspensions were centrifuged at 10,000 ×*g* for 10 min and filtered (0.2 μm, 25 mm, Millipore, Billerica, MA, USA). To monitoring its effect on the growth of bacteria, the fecal extract was diluted in MBH medium to a final concentration of 1.0% or 2.5%. Fecal samples were collected from one human volunteer on three occasions. The use of human fecal samples was approved by the FDA Research Involving Human Subjects Committee (approval number 09-033T). Six separate dilutions of 0.1, 0.2, 0.3, 0.5, 1 and 5 µg/mL enrofloxacin were prepared in sterile water. All solutions were stored in the refrigerator until use. 

### 3.2. Growth Kinetics of *S. enterica* and *L. monocytogenes*

The MIC of enrofloxacin for *S. enterica* and *L. monocytogenes* was verified in a 96-well microtiter plate according to the method described by CLSI [27]. Serial dilutions of enrofloxacin, in concentrations ranging from 0.01 to 0.5 μg/mL, were prepared in 200 μL of MHB medium. The wells were inoculated with 2 × 10^5^ CFU/mL of each bacterium and the plate was incubated for 24 h at 37 °C. The MIC of the enrofloxacin, in which no bacteria survived, was then determined. The influence of addition of sucrose and fecal extract on the effect of enrofloxacin on the kinetics of growth of *S. enterica* and *L. monocytogenes* was measured by the addition of 5 mM sucrose and 1 and 2.5% sterilized fecal extract to the MHB medium as previously described [12]. Briefly, 200 µL of each of the media containing different additives was added to duplicate wells of 96-well plates. Dilutions of 0.01, 0.02, 0.03, 0.05, 0.08, 0.1 and 0.5 μg/mL of enrofloxacin were made in each of the media containing different additives. *S. enterica* and *L. monocytogenes* cells (2 × 10^5^ CFU/mL) were added to each of the wells. The microtiter plates were directly incubated in a Synergy MX spectrophotometer (BioTek Instruments, Winooski, VT, USA). For the second passage, equivalent OD of the cells were transferred from the well containing 0.01 μg/mL of enrofloxacin to the 2nd microtiter plates prepared as described for the first passage. Duplicate control wells, containing media without antibiotics and with and without additives, were also inoculated with the same quantities of cells. For the third passage, bacteria grown with 0.01 μg/mL of enrofloxacin in the second passage were used. These experiments were repeated for three times. Statistical analyses were performed using a one-way analysis of variance, with a *p* value of <0.05 being considered significant.

### 3.3. Quinolone Resistance Determining Region (QRDR) and Pulsed-Field Gel Electrophoresis (PFGE)

For the detection of mutations in the QRDR of gyrA, DNA was extracted from *S. enterica* cell of 96 wells. These included the wells of control cells without any treatment and those grown with 0.02, 0.03, 0.05, 0.08, 0.1 and 0.5 μg/mL of enrofloxacin and with and without additives from the second and third passages. QIAamp DNA Micro Kit from (Qiagen Inc., Valencia, CA, USA) was used for DNA extraction. PCR amplification of the QRDR of gyrA was carried out by using primers for *S. enterica*, GYRAF (5'-CGTTGGTGACGTAATCGGTA-3') and GYRAR (5'-CCGTGCCGTCATAGTTATCA-3') [12,28]. QRDR amplicons from *S. enterica* grown in MHB with and without 2.5% fecal extract and enrofloxacin were sequenced and compared [12]. 

Total 24 genomic DNA samples from 0.06 μg/mL of enrofloxacin and with and without 2.5% sterilized human fecal extract were selected in the first, second and third passages. Control genomic DNA samples were selected containing media without antibiotics and with and without 2.5% fecal extract. These samples were subjected to PFGE after restriction digestion of *S. enterica* with AvrII and XbaI and *L. monocytogenes* with ApaI and AscI [29]. DNA plugs were digested with 20 U of restriction enzyme (New England Biolabs, Ipswich, MA, USA) at 37 °C for 5 h. The digested DNA was separated on 1.0% SeaKem Gold (FMC Corp., Philadelphia, PA, USA) agarose gels with a Chef-Mapper III PFGE (BioRad Laboratories, Hercules, CA, USA) system for 20 h as described earlier [29]. The gels were stained for 30 min with ethidium bromide (EtBr), destained with distilled water, and photographed using the Eagle Eye II gel documentation system. The interpretation of PFGE patterns was analyzed manually.

### 3.4. Effect of Efflux Pump Inhibitors on Enrofloxacin Sensitivity and EtBr Accumulation in *S. enterica*

The effects of efflux pump inhibitors on the sensitivity of bacteria to enrofloxacin were examinedby adding reserpine (Aldrich, Milwaukee, WI, USA) or carbonyl cyanide-*m*-chlorophenylhydrazone (CCCP, Sigma, St. Louis, MO, USA). The final concentrations of reserpine and CCCP were 33 µM and 10 µM, respectively [12,23,24].

The accumulation of EtBr was measured in the presence and absence of the efflux pump inhibitors reserpine and CCCP in the bacteria that were grown for three passages in the media with and without 2.5% fecal extract as previously described [30]. Samples of *S. enterica*, which were grown for 24 h and 48 h in MHB medium with or without 2.5% fecal extract, in the presence and absence of 0.01 and 0.08 µg/mL of enrofloxacin, were used for the assays in black 96-well plates. One percent EtBr was added to each well containing the samples. The accumulation of EtBr was monitored using excitation and emission wavelengths of 530 nm and 600 nm, respectively, by a SpectraMAX Gemini XS mass spectrometer (Molecular Devices, Sunnyvale, CA, USA). For the effect of inhibitors on the EtBr accumulation, 10 µM CCCP or 33 µM reserpine was added to duplicate wells, at the beginning of the experiments or 4 min thereafter, in separate and similar experiments.

### 3.5. Negative Staining and Thin Sectioned Transmission Electron Microscopy (TEM)

Cell morphology was observed by transmission electron microscopy (TEM) after negative staining with 2% uranyl acetate. Cells were primary-fixed for 24 h in 4% glutaraldehyde in phosphate buffer. Post-fixation was carried out in 1% osmium tetroxide buffer for 1 h followed by dehydration in a graded ethanol series and embedding in an Epon/Araldite mixture. Thin sections (70 nm) were obtained using a diamond knife equipped Leica EM UC6 ultramicrotome. Thin sections were collected on 100 mesh copper grids and then stained using a Leica EM AC20 grid-stainer and uranyl acetate (Leica Ultrostain I) for 15 min, followed by lead citrate (Leica Ultrostain II) for 4 min and final de-ionized water wash. Micrographs were taken at 80 kV with a JEOL-2100 TEM [31]. 

### 3.6. Comparison of Fatty Acids of *S. enterica* and *L. monocytogenes*

The extraction and analysis of fatty acids from *S. enterica* and *L. monocytogenes* were done as previously described [12]. Approximately 60 mg of cell mass was harvested from the third passage bacteria grown with and without 0.06 μg/mL of enrofloxacin in the presence and absence of 2.5% fecal extract. Cells harvested were stored in the refrigerator until analysis. An Agilent series 6,890 GC was used for the analysis of fatty acid methyl esters using the SHERLOCK Microbial Identification System (MIDI).

## 4. Conclusions

Fecal extract enhanced the ability of *S. enterica* and *L. monocytogenes* to grow at higher concentrations of enrofloxacin in the first and subsequent passages. The nature of the effect of fecal extract on the alteration of bacterial susceptibility to enrofloxain is not known. The effects may be related to the alteration of cell permeability and transport, as evident by the changed in the bacterial lipid composition noted in the proportion of unsaturated and saturated fatty acids in *S. enterica* and *L. monocytogenes*. This may have affected the transport as evident by the effect of efflux inhibitors on drug susceptibility, increases in the bacterial tolerance to the chemical osmotic pressure. The effect could also be the result of unknown molecules in the fecal extract that affected bacterial interaction with the drug and resulted in other changes that affect bacterial sensitivity and growth.
